# Herbal Remedies for Acne Vulgaris from the Viewpoint of Persian Medicine

**Published:** 2020-05

**Authors:** Rasoul SHAKIBA, Bagher MINAII, Jale ALIASL, Mahdad SHAKIBA, Roshanak GHODS

**Affiliations:** 1. Research Institute for Islamic and Complementary Medicine, Iran University of Medical Sciences, Tehran, Iran; 2. School of Persian Medicine, Tehran University of Medical Sciences, Tehran, Iran; 3. Department of Histology, School of Medicine, Tehran University of Medical Sciences, Tehran, Iran; 4. Traditional Medicine Clinical Trial Research Center, Shahed University, Tehran, Iran; 5. School of Pharmacy, International Campus, Tehran University of Medical Sciences, Tehran, Iran

## Dear Editor-in-Chief

Acne vulgaris is a chronic inflammatory disorder of pilosebaceous follicles (1). It is the most common skin problem in the world, affecting nearly 80% to 85% of individuals between 12 and 25 years. of age (2). Clinically acne mainly manifests in the form of comedones, papules, pustules and nodules (1). The major pathogenic factors in acne lesions including follicular hyperkeratinization, increase of sebaceous gland secretion, Propionibacterium acnes colonization and inflammation (1, 3). Acne can cause scarring, as well as psychological disorders such as decreased self-esteem, depression, anxiety and even suicide (3). Nowadays, systemic antibiotics, isotretinoin and topical drugs are used for treatments of severe acne (4). Actually, antibiotics and keratolytics affect the pilosebaceous unit, but many patients are not completely satisfied due to their complications (5). Although isotretinoin is an effective treatment for acne vulgaris, it has some common adverse effects (5) there is also a high level of antibiotics resistance (4), therefore, consumption of complementary and traditional medicine such as herbal drugs are increasing and common amongst patients with acne and other disease, because people believed medicinal plants have low adverse effects and many plants seem to have inhibitory effects on the growth of bacteria, fungi and viruses in vitro, however. There are some clinical evidence about the effectiveness and safety of these herbs in the treatment of acne and other skin infections(6). Although the term which is exactly equivalent to acne is not mentioned as a specific disease in Persian medicine resources, according to the symptoms and signs of acne in medical textbooks, clinical manifestations similar to acne have been considered under the topic of *Awram* and *Bothur* (swelling and rashes) in Persian medicine sources (7)

Based on the principles of Persian medicine, *Awram* and *Bothur* occur as a result of humoral imbalances and the body inability to eliminate undesirable and unwanted substances through common and logical ways (7), therefore, accumulated materials are driven out of the body through other ways like the skin, which can cause various skin problems. In this study, the reference books of Persian medicine such as *Makhzan ol advieh* (8), *Canon of Medicine* (9)and *Exir-e Azam* (10) were reviewed for recommended drugs to treatment of *Awram* and *Bothur*, most of these medications are resolvent (*Mohallil*) and have dissolving property. Resolvent drugs which are capable of producing heat can remove abnormal humor from the place accumulated by vaporizing properties (8). Some of them include cedar, sandalwood, vinegar, coriander, barley flour, bean flour, fenugreek, spurge, peganum, sweet clover, chamomile, flax, psyllium (8). Medicinal plants that used most commonly were introduced in Table 1.

**Table 1:** Some medicinal plants suggested for swelling and rashes in Persian medicine sources

	***Common name***	***Persian medicine name***	***Scientific name***	***Family***
1	Christ's thorn jujube	Cedr	Ziziphus spina-christi	Rhamnaceae
2	Sweet clover	Eklilolmalek	Melilotus officinalis	Fabaceae
3	Chamomile	Babooneh	Matricaria chamomilla	Asteraceae
4	Myrtle	Moord	Myrtus communis	Myrtaceae
5	Marigold	Hamisheh bahar	Calendula officinalis	Compositae
6	Myrrh	Morr	Commiphora molmol	Burseraceae
7	Marshmallow	Khatmi	Althaea officinalis	Malvaceae
8	Henna	Hana	Lawsonia inermis	Lythraceae
9	Barley	Jo	Hordeum distichon	Poaceae
10	Broad bean	Baghella	Vicia faba	Fabaceae
11	Fenugreek	Shanbalileh	Trigonella foenum-graecum	Fabaceae

According to Persian medicine, there are a lot of medicinal herbs which recommended for treatment of swelling and rash, which also can be used in acne vulgaris.

It is particularly interesting to more evaluation of these drugs on acne vulgaris for clarification of their efficacy and therapeutic effects which may help to find new sources for treatment of acne.
